# Natural temperature fluctuations promote *COOLAIR* regulation of *FLC*

**DOI:** 10.1101/gad.348362.121

**Published:** 2021-06

**Authors:** Yusheng Zhao, Pan Zhu, Jo Hepworth, Rebecca Bloomer, Rea Laila Antoniou-Kourounioti, Jade Doughty, Amelie Heckmann, Congyao Xu, Hongchun Yang, Caroline Dean

**Affiliations:** John Innes Centre, Norwich Research Park, Norwich NR4 7UH, United Kingdom

**Keywords:** *FLC*, *COOLAIR*, noncoding RNA, vernalization, temperature-sensing

## Abstract

Plants monitor many aspects of their fluctuating environments to help align their development with seasons. Molecular understanding of how noisy temperature cues are registered has emerged from dissection of vernalization in *Arabidopsis*, which involves a multiphase cold-dependent silencing of the floral repressor locus *FLOWERING LOCUS C* (*FLC*). Cold-induced transcriptional silencing precedes a low probability PRC2 epigenetic switching mechanism. The epigenetic switch requires the absence of warm temperatures as well as long-term cold exposure. However, the natural temperature inputs into the earlier transcriptional silencing phase are less well understood. Here, through investigation of *Arabidopsis* accessions in natural and climatically distinct field sites, we show that the first seasonal frost strongly induces expression of *COOLAIR*, the antisense transcripts at *FLC*. Chamber experiments delivering a constant mean temperature with different fluctuations showed the freezing induction of *COOLAIR* correlates with stronger repression of *FLC* mRNA. Identification of a mutant that ectopically activates *COOLAIR* revealed how *COOLAIR* up-regulation can directly reduce *FLC* expression. Consistent with this, transgenes designed to knockout *COOLAIR* perturbed the early phase of *FLC* silencing. However, all transgenes designed to remove *COOLAIR* resulted in increased production of novel convergent *FLC* antisense transcripts. Our study reveals how natural temperature fluctuations promote *COOLAIR* regulation of *FLC*, with the first autumn frost acting as a key indicator of autumn/winter arrival.

## Abstract (precis)

In this study, Zhao et al. set out to characterize how plants respond to cold through regulation of FLC expression. Using genetics and genomics approaches, the authors reveal how natural temperature fluctuations promote COOLAIR regulation of FLC, with the first autumn frost acting as a key indicator of autumn/winter arrival.

As sessile organisms, plants have to extract specific temperature cues from fluctuating environments to time their developmental transitions. Knowledge of these mechanisms will be key to understanding the consequences of climate change. In *Arabidopsis*, a major determinant of seasonal flowering is the floral repressor *FLC*, which is epigenetically silenced by prolonged cold during vernalization (Michaels and Amasino 1999; Sheldon et al. 2000). Vernalization involves a multiphase cold-dependent silencing, where cold-induced transcriptional silencing precedes a low probability PRC2 epigenetic switching mechanism. The epigenetic switch is promoted by the PRC2-accessory protein VIN3, which is induced slowly, taking weeks to reach maximal levels (Sung and Amasino 2004). VIN3 accumulation requires absence of warm temperature spikes, in addition to long-term cold exposure (Hepworth et al. 2018; Zhao et al. 2020). The transcriptional silencing involves cold-induced *FLC* antisense transcripts, *COOLAIR,* whose induction correlates with *FLC* transcriptional shutdown and switching of the chromatin states at *FLC* (Swiezewski et al. 2009; Csorba et al. 2014; Rosa et al. 2016). *COOLAIR* also functions to promote rapid cycling in plants in warm conditions in a cotranscriptional chromatin silencing mechanism that links promotion of proximal polyadenylation to *FLC* histone H3K4me1 demethylation (Costa and Dean 2019; Fang et al. 2020; Wu et al. 2020).

*COOLAIR* is highly conserved across different species (Castaings et al. 2014; Hawkes et al. 2016; Jiao et al. 2019) and has been shown to contribute to the variation in *FLC* regulation across natural *Arabidopsis* accessions (Shindo et al. 2006; Coustham et al. 2012; Li et al. 2014). Notably, a noncoding single-nucleotide polymorphism (SNP) alters *COOLAIR* splicing, increasing *FLC* transcription levels (Li et al. 2015). This mechanism involves the activator FRIGIDA protein and distally polyadenylated *COOLAIR* (Johanson et al. 2000; Geraldo et al. 2009; Li et al. 2018). The natural variation studies implicating a functional role for *COOLAIR* in *FLC* regulation have been followed up by transgene experiments designed to further explore the mechanism of *COOLAIR* action (Csorba et al. 2014; Wang et al. 2014; Rosa et al. 2016). However, in some cases, studies of transgenes aimed at attenuating antisense expression have concluded that *COOLAIR* expression is not required for vernalization (Helliwell et al. 2011; Li et al. 2018; Luo et al. 2019; Luo and He 2020). This led to the suggestion that *COOLAIR* functions in *FLC* regulation at warm temperatures but potentially not in the cold. In order to test this, we investigated the role of *COOLAIR* in vernalization, in both natural and laboratory conditions and in different accessions. Our study demonstrates the importance of *COOLAIR*-mediated *FLC* silencing in natural conditions, with the first seasonal freezing temperatures leading to *COOLAIR*-mediated *FLC* transcriptional silencing.

## Result

### *COOLAIR* shows variable temperature sensitivity and is highly up-regulated by freezing temperatures

Through the analysis of >1000 worldwide *Arabidopsis* natural accessions, we previously identified five predominant *FLC* haplotypes defined by noncoding SNPs (Li et al. 2014). According to the varied vernalization responses measured by flowering time after growth at a constant 5°C, these haplotypes were classified into “rapid vernalizing” (RV) and “slow vernalizing” (SV) types (Li et al. 2014). To further investigate their vernalization response in field conditions, we selected representative accessions of these five major haplotypes (RV: Edi-0 and Col *FRI*^*SF2*^; SV: Var2-6, Ull2-5, and Bro1-6), as well as an extra SV accession, Löv-1, collected from the North Swedish field site (Duncan et al. 2015; Qüesta et al. 2020). To allow the comparison among these haplotypes, we generated new near-isogenic lines (NILs) by repeatedly backcrossing each *FLC* haplotype to Col *FRI*^*SF2*^, our reference genetic background (“Col *FRI*”) (Duncan et al. 2015; Li et al. 2015). Recent work has shown that these accessions and their associated NILs show variation in response to autumn cold in the field (Hepworth et al. 2020).

Here, we analyzed *COOLAIR* induction in field conditions to determine whether *COOLAIR* plays a role in natural conditions in different *Arabidopsis* accessions and their associated NILs. Three climatically different field sites were chosen: Norwich, United Kingdom (52° 62.2191′ N, 1°22.1695´ E; temperate oceanic); Ullstorp, Sweden (56°06.6721′ N, 13°94.4655′ E; “South Sweden”; a warm-summer continental); and Ramsta, Sweden (62°50.988′ N, 18°11.570′ E; “North Sweden”; subarctic) (Supplemental Figs. S1, S2; Antoniou-Kourounioti et al. 2018; Hepworth et al. 2018, 2020). Norwich generally has mild winters with few frosts, while in Ullstorp temperatures often fall below freezing, whereas the Ramsta site is usually snow-covered during part of the winter. The experiments ran from late summer/early autumn 2014 until spring 2015 and were repeated in North Sweden from late summer 2016 to the spring of 2017. Sowing times were adjusted to each site (earlier further north), and in the first year, two plantings were performed in North Sweden, a fortnight apart.

Three SV accessions (Löv-1, Ull2-5, Bro1-6) showed higher *COOLAIR* levels than the RV Col *FRI* across all the plantings (Supplemental Fig. S2). In contrast, Col-0 (which is a rapid cycler with no vernalization requirement for flowering) had very low levels of total *COOLAIR* in Norwich and at most time points in Sweden. *COOLAIR* levels in the RV accession Edi-0 and its NIL were very similar to Col *FRI* in Norwich, but in Sweden, especially North Sweden, *COOLAIR* was higher in Edi-0 than Col *FRI.* This was also the case for Var2-6, with early differences only in Sweden. These data suggest *COOLAIR* induction in the different accessions shows different temperature sensitivity.

**Figure 1.**
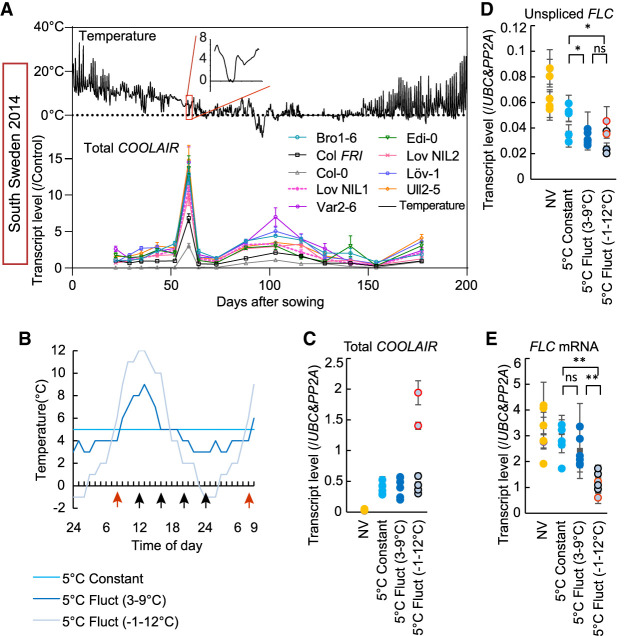
*COOLAIR* expression is highly induced by freezing temperature. (*A*) *COOLAIR* expression in all genotypes in a field in South Sweden over winter 2014–2015. Plots in the *top* panel show the temperature profile in the field with the first appearance of freezing temperature highlighted and expanded in the red box. Plots in the *bottom* panel show the relative transcript level of *COOLAIR* as analyzed by RT-qPCR. (*B*) The temperature profiles of chambers set up to analyze the interrelationship of freezing, *COOLAIR* expression, and *FLC* expression. Plants grown in these chambers were used to generate data shown in *C–E*. (*C–E*) Relative transcript level of *COOLAIR* (*C*), unspliced *FLC* (*D*), and *FLC* mRNA (*E*) measured throughout the day (NV [nonvernalized]) and after 2 wk of different cold exposure. Results were presented by combining the data of the six sampling points from each treatment. The sampling times are indicated by arrows in *B*. More details are described in the Materials and Methods. (*C*) *COOLAIR* data points in red (indicated by the red arrow in *B*) were those taken ∼8 h after freezing. Vernalized treatments compared by ANOVA with Tukey's post hoc test. (*) *P* < 0.05, (**) *P* < 0.01, (ns) no significance. Expression data were normalized as indicated in each panel. Error bars show SEM.

We noticed a strong peak of expression of *COOLAIR* in all genotypes, even Col-0, in the second week of measurements (the 26th day after sowing) in the first North Sweden planting and the sixth week (the 59th day after sowing) in the South (Fig. 1A; Supplemental Figs. S1, S2). These unusual peaks occurred when temperatures dipped below 0°C on the morning of the day of measurement (e.g., for South Sweden 2014, see Fig. 1A; for North Sweden 2016, see Supplemental Fig. S3A,B).

### Recapitulation of the *COOLAIR* expression spike in temperature-controlled chambers

We confirmed the up-regulation of *COOLAIR* by freezing by reproducing the temperature profile of the week before the first peak in South Sweden in a growth cabinet. *COOLAIR* expression rose within an hour of experiencing freezing and peaked ∼8 h after freezing but returned to cool-temperature levels within 24 h (Supplemental Fig. S3C). There was a small reduction in the level of *FLC* transcripts immediately after freezing exposure (Supplemental Fig. S3D,E), reflecting the coordination of antisense–sense transcriptional circuitry. To further analyze the interrelationship of freezing, *COOLAIR* expression, and *FLC* expression, we grew seedlings for 2 wk in matched chambers at an average temperature of 5°C, but given either as a constant temperature or two different fluctuating temperature regimes repeated every 24 h (Fig. 1B). We saw the expected induction of proximal polyadenylation of *COOLAIR* by constant cold; however, this was significantly enhanced in the chamber where the fluctuations included a below-freezing period (Fig. 1C; Supplemental Fig. S3F–I). Of note, this hyperinduction of *COOLAIR* is not just a consequence of a steep temperature drop, as *COOLAIR* induction was lower after transfer from nonvernalized (NV) 20°C to constant 5°C (Supplemental Fig. S3J). All the temperature regimes led to a reduction in *FLC* expression, including the *FLC* mRNA and the unspliced transcript level (nascent transcript containing intron 2 and intron 3) used as a proxy for transcription (Fig. 1D,E). However, the enhanced *COOLAIR* induction in the freezing regime caused significantly lower *FLC* mRNA levels (Fig. 1E). The mechanism behind the different behavior of the unspliced transcripts and mRNA after exposure to freezing is currently unclear.

### Genetic up-regulation of *COOLAIR* is associated with *FLC* transcriptional shutdown

**Figure 2.**
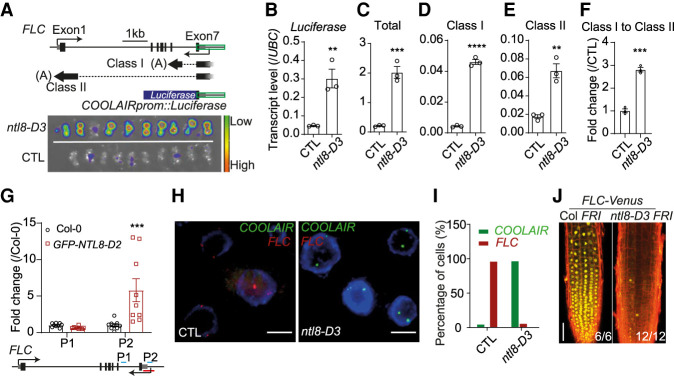
Genetic up-regulation of *COOLAIR* expression is associated with *FLC* transcriptional reduction. (*A*) Luminescence assay for warm-grown *ntl8-D3* plants (20°C). (CTL) Progenitor line carrying transgenic *COOLAIRprom::luciferase* reporter. A diagram of the *COOLAIRprom::luciferase* reporter is shown in the *top* panel. Untranslated region (UTR) of *FLC* is indicated by a gray box, and exons are represented by black boxes. A green box indicates the *COOLAIR* promoter. Simplified diagrams are included to show the alternative polyadenylation of *COOLAIR*: proximal polyadenylated class I and distal polyadenylated class II. (Black solid line) Exons, (black dashed line) introns, (gray lines) transcriptional start region. (*B–E*) qPCR analyzing *luciferase* (*B*), total *COOLAIR* (*C*), class I *COOLAIR* (*D*), and class II *COOLAIR* (*E*) transcript levels in warm conditions (20°C) for CTL and *ntl8-D3*. Levels normalized to *UBC*. Error bars show SEM of three biological replicates. (*F*) Ratio of proximal class I to distal class II *COOLAIR* with the data from *C*–*E*, normalized to CTL. Unpaired two-tailed *t*-test was performed, and significances for each comparison are shown. (**) *P* < 0.01, (***) *P* < 0.001, (****) *P* < 0.0001. (*G*) ChIP analysis of NTL8 binding at *COOLAIR* region. (Control) Col-0. Error bars show SEM of eight replicates. Two-way analysis of variance (ANOVA) with Turkey's multiple comparisons test was performed, and significances for individual comparison of interest are shown. (***) *P* < 0.001. Positions of amplicon P1 and P2 are indicated on the diagram. (*H*) Representative images of nuclei (indicated by DAPI staining; blue) hybridized with intronic smRNA FISH probes for *COOLAIR* (green) and *FLC* (red) showing mutually exclusive transcription in CTL and *ntl8-D3* mutants. Plants were grown at 20°C. Scale bars, 5 μm. (*I*) Percentage of cells with *FLC* or *COOLAIR* signal in nonprevascular cells in CTL (*n* = 125 for *FLC*, 207 for *COOLAIR*) and *ntl8-D3* (*n* = 171 for *FLC*, 269 for *COOLAIR*). (*J*) Imaging of FLC-Venus in Col *FRI* and *ntl8-D3 FRI* grown at 20°C. Numbers of independent roots assayed for each genotype are indicated in the *bottom right* corner. Scale bar, 50 μm.

The complex relationship of the *FLC* down-regulation and the freezing-induced *COOLAIR* spike was intriguing with respect to the registration of external temperature and *FLC* regulation. In order to better understand the relationship between *COOLAIR* up-regulation and *FLC* repression, we sought to identify a mutant constitutively expressing high levels of *COOLAIR*. A forward genetic screen using a *COOLAIRprom::luciferase* reporter (Swiezewski et al. 2009; Sun et al. 2013), identified a dominant mutant, *ntl8-D3*, that had a high luciferase signal in warm conditions (Fig. 2A,B). All endogenous *COOLAIR* transcripts, including both proximal (class I) and distal (class II) polyadenylated forms, showed ectopic up-regulation in *ntl8-D3* (Fig. 2C–E), with a relative increase in proximal polyadenylation as seen in wild-type plants after cold (Fig. 2F; Supplemental Fig. S3I). Two separate mutant alleles, *ntl8-D1* and *ntl8-D2,* and three overexpression transgenics, *ntl8-OE1*, *ntl8-OE2*, and *ntl8-OE3*, showed the same *COOLAIR* up-regulation (Supplemental Fig. S4A–F; Zhao et al. 2020). *ntl8-D* alleles cause a truncation in the NTL8 protein that deletes the C-terminal transmembrane domain and results in enhanced nuclear localization (Supplemental Fig. S4G; Zhao et al. 2020). NTL8 encodes a NAC domain transcription factor that directly binds *VIN3* and the *COOLAIR* promoter (Fig. 2G; Supplemental Fig. S4H; O'Malley et al. 2016; Xi et al. 2020; Zhao et al. 2020). Interestingly, *the ntl8-D* mutant alleles are also defective in long-term temperature regulation of *VIN3* (Zhao et al. 2020), and the same logic that we have described for VIN3 accumulation—namely, reduced dilution of wild-type NTL8 protein during cold exposure—may be important in promoting long-term *COOLAIR* expression in autumn conditions. Previous analysis of *ntl8-D2 vin3-6* double mutants revealed that the *VIN3* overexpression, which also occurs in the *ntl8-D* mutant, was not necessary for the lower *FLC* expression before vernalization (Zhao et al. 2020). In order to better understand whether the higher *COOLAIR* expression was a reflection of more expression from the same cells or a higher fraction of cells expressing *COOLAIR*, we performed single-molecule RNA fluorescence in situ hybridization (smRNA FISH) on the *ntl8-D3* mutant in warm conditions (Rosa et al. 2016). *COOLAIR* expression was found at high levels in all cells of the root (Fig. 2H,I); thus, *ntl8-D3* had expanded the expression zone normally seen for *COOLAIR*, from just prevasculature cells to all cell types (Rosa et al. 2016). This is consistent with the reduced FLC-Venus signals in the *ntl8-D3* mutant in warm conditions (Fig. 2J). This mutually exclusive transcription of *COOLAIR* and *FLC* supports the repressive effect of *COOLAIR* on *FLC* transcription from the same gene copy, agreeing with the previous study (Rosa et al. 2016).

### *COOLAIR* is required for *FLC* transcriptional shutdown in natural and laboratory conditions

**Figure 3.**
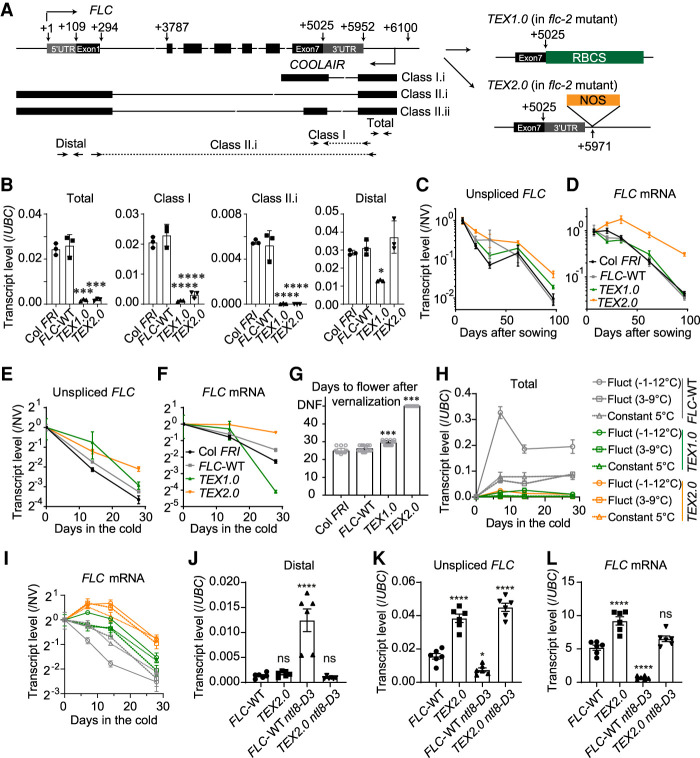
*COOLAIR* is required for *FLC* regulation in both field and laboratory conditions. (*A*) Schematic illustration of wild-type *FLC*, *TEX 1.0*, and *TEX 2.0*. Untranslated region (UTR) of *FLC* is indicated by gray box, and exons are represented by black boxes. Black arrows indicate positions of primers for detecting *COOLAIR* (the detailed positions are distal, 40–135; class I, 5634–5953; total, 5792–6034). (*B*) Antisense transcript levels in nonvernalized (NV) wild-type reference Col *FRI*, wild-type transgenic control (*FLC*-WT), and two *TEX* lines. Levels normalized to *UBC*. Error bars show SEM of three biological replicates. (*C*,*D*) Unspliced *FLC* transcripts (*C*) and *FLC* mRNA (*D*) level in wild-type and *TEX* lines in the Norwich field in 2016–2017. Expression normalized first to an internal control (see the Materials and Methods) and then to the initial level (time point 1) of each genotype. Error bars show SEM. Data of the Col *FRI* were previously reported (Antoniou-Kourounioti et al. 2018). (*E*,*F*) Unspliced *FLC* transcripts (*E*) and *FLC* mRNA (*F*) for the wild-type and two *TEX* lines under constant laboratory vernalization conditions (5°C). Transcript level was normalized first to an internal control and then to nonvernalized (NV; 20°C). Error bars show SEM of three biological replicates. (*G*) Flowering time phenotype of wild-type and two *TEX* lines after 4 wk of cold in laboratory conditions. (UNF) Not flowering after 50 d, *n* = 20 plants for each genotype. (*H,I*) Plants were grown in conditions shown in Figure 1B. Relative transcript level of total *COOLAIR* (*H*) and *FLC* mRNA (*I*) was measured ∼8 h after freezing and after 7, 14, and 28 d of cold exposure. Levels were normalized first to *UBC* and then to the NV of each genotype. Error bars show SEM of two biological replicates for the 7-d time point, and three biological replicates for all other time points. (*J–L*) qPCR analysis of distal *COOLAIR* (*J*), unspliced *FLC* (*K*), and *FLC* mRNA (*L*) transcript levels in warm conditions in *FLC*-WT, *TEX2.0*, *FLC*-WT *ntl8-D3*, and *TEX2.0 ntl8-D3* mutants. Levels were normalized to *UBC*. Errors show SEM of six biological replicates. One-way analysis of variance (ANOVA) with ’Dunnett's multiple comparisons test was performed, and significances for individual comparisons of interest are shown. (*) *P* < 0.05, (***) *P* < 0.01, (****) *P* < 0.001, (ns) no significance.

We then investigated how *COOLAIR* may mediate *FLC* shutdown by interrogating transgenic lines carrying *FLC* disrupted in production of *COOLAIR* (Terminator Exchange 1.0 and 2.0, *FLC::FLC-TEX1.0* and *FLC*-*TEX2.0*). The *TEX1.0* line was generated in a previous study (Csorba et al. 2014), and the *TEX2.0* line was newly generated by inserting a NOS terminator to terminate *COOLAIR* transcription without disturbing the 3′ UTR of *FLC* (Fig. 3A). *COOLAIR* transcripts (detected by primers for total, class I, and class II.i) (Csorba et al. 2014) were greatly reduced in both the *TEX1.0* and *TEX2.0* lines (Fig. 3B). In natural conditions, the unspliced *FLC* transcript levels in both *TEX1.0* and *TEX2.0* reduced more slowly than in wild-type controls (transgenic *FLC*-WT and nontransgenic Col *FRI*) during vernalization (Fig. 3C), supporting a role for *COOLAIR* in mediating *FLC* transcriptional shutdown. Moreover, the *FLC* mRNA level was accordingly higher in *TEX2.0* but not in *TEX1.0* (Fig. 3D), likely due to the change in the 3′ UTR causing instability of the *FLC* mRNA in *TEX1.0* (Csorba et al. 2014). Consistently, the same *FLC* expression in *TEX1.0* and *TEX2.0* and late-flowering phenotypes were also observed in laboratory conditions (Fig. 3E–G), reinforcing the view that *COOLAIR* plays a role in *FLC* transcriptional shutdown.

Given that the freezing-enhanced *COOLAIR* induction led to significantly lower *FLC* mRNA levels (Fig. 1E), these two *TEX* lines were also tested in the temperature regimes of Figure 1B. Similar to the observation in Figure 1, *COOLAIR* was more induced in wild-type plants in the freezing regime compared with the other two temperature regimes (Fig. 3H), and *FLC* mRNA levels in the freezing regimes were accordingly lower (Fig. 3I). In contrast, such lower *FLC* mRNA levels associated with freezing temperature were not observed in either of the *TEX* lines, where *COOLAIR* induction is greatly reduced (Fig. 3H,I). This supports the view that *COOLAIR* is responsible for the enhanced decrease in *FLC* mRNA levels in the freezing regimes. To further determine the causality of *COOLAIR* up-regulation and *FLC* down-regulation, we generated a double mutant of *ntl8-D3* with *TEX2.0*, as well as a double mutant of *ntl8-OE3* with a *COOLAIR* promoter deletion line, *FLC_ΔCOOLAIR_* (Luo et al. 2019). In both double mutants, there is no longer *COOLAIR* up-regulation by NTL8 (Fig. 3J; Supplemental Fig. S4I–L). We found that *FLC* down-regulation in *ntl8-D3* and *ntl8-OE3* is substantially suppressed by the *COOLAIR* knockout (Fig. 3K,L; Supplemental Fig. S4M). Therefore, *COOLAIR* up-regulation is a major causal factor for the *FLC* down-regulation in the *ntl8-D* or *ntl8-OE* mutants, supporting a direct role of *COOLAIR* or *COOLAIR* transcription in *FLC* transcriptional shutdown.

### Transgenes aimed at knocking out *COOLAIR* promote production of novel convergent antisense transcripts

**Figure 4.**
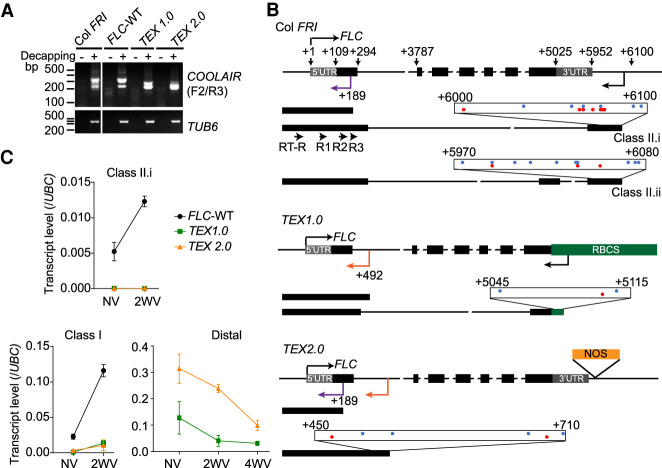
Removal of *COOLAIR* promotes intragenic convergent antisense transcripts in *TEX* lines. (*A*) Agarose gel showing the antisense transcripts detected by 5′ RACE in wild-type reference Col *FRI*, wild-type transgenic control (*FLC*-WT), and two *TEX* lines after 2 wk of cold (5°C). *TUB6* was used as a control. Samples without (−) or with (+) decapping treatment are indicated. Primers used are mapped in *B*. (*B*) Schematic illustrations showing the antisense transcriptional start sites (TSSs) mapped by 5′ RACE in the wild-type and *TEX* lines. The untranslated region (UTR) of *FLC* is indicated by a gray box, and exons are represented by black boxes. Black arrows show the positions of *COOLAIR* TSS in the wild type and *TEX 1.0*. Purple arrows show the positions of *CAS* TSS common in the wild type and *TEX 2.0*, while orange arrows show that of *CAS* TSSs only in *TEX* lines. Antisense TSSs were mapped in the scaled boxes with red dots representing those in NV samples and blue dots representing those in 2WV samples. Numbers indicate distance (in base pairs) from the *FLC* TSS. Primers (RT-R, R1, R2, and R3) used for 5′ RACE are indicted in the Col *FRI* schematic. (*C*) Expression level of antisense transcripts in NV and cold-treated wild-type and *TEX* lines. (2WV) Two weeks of cold treatment; (4WV) 4 wk of cold treatment. Error bars show SEM for three biological replicates. Primers used are illustrated in Figure 3A.

During the analysis of the *TEX* lines, it became clear that distally polyadenylated *COOLAIR* was still produced (Fig. 3B). This was unexpected given the presence of the upstream terminator sequence. To understand the origin of the *COOLAIR* distal amplicon in the *TEX1.0* and *TEX2.0* lines, 5′ RACE (rapid amplification of cDNA ends) was performed (Supplemental Fig. S5A). In wild-type plants, grown without (NV) or with 2 wk of cold treatment (2WV), two major spliced distal *COOLAIR* forms with a range of transcriptional start sites were identified (Fig. 4A,B), consistent with the previous study (Swiezewski et al. 2009). In addition, a low abundance convergent antisense transcript (hereafter referred to as *CAS* as previously defined by Kindgren et al. [2020]) possessing a 5′ cap was identified with a transcriptional start site within *FLC* exon 1 (Fig. 4A,B; Supplemental Table S1). In the *TEX1.0* line, antisense transcription was found to initiate within the *RBCS* terminator fragment, producing a distal *COOLAIR* with the same splice sites as on the endogenous locus (Fig. 4A,B; Supplemental Table S1). In the *TEX 2.0* line, no *COOLAIR* transcripts were detected except for the *CAS* transcript initiating within *FLC* exon 1 (Fig. 4A,B). Interestingly, additional *CAS*s containing a 5′ cap were identified in both *TEX* lines originating from different positions inside *FLC* intron 1 (Fig. 4A,B; Supplemental Table S1). The multiple transcriptional start sites of *CAS* (Fig. 4B) are likely to contribute to the higher antisense expression in *TEX2.0* (Figs. 3B, 4C). The proximally polyadenylated *COOLAIR* (class I) present at very low levels in both *TEX* lines is cold induced (Figs. 3B, 4C) and originates from the residual *FLC* fragment in the *flc-2* background, rather than the *TEX* transgene (Supplemental Fig. S5B). Thus, the major *COOLAIR-CAS* transcripts in the *TEX* lines are novel *CAS* and are not cold induced (Fig. 4C). Given the low frequency of *CAS* in the wild type, their transcription is likely to be suppressed by *COOLAIR* transcription from the upstream native promoter.

We further investigated three other transgenic lines (*FLC + MAF2-T*, *FLC + NOS-T*, and *FLC_ΔCOOLAIR_*) that had been designed to remove *COOLAIR* from *FLC* (Li et al. 2018; Luo et al. 2019). The *FLC + MAF2-T* and the *FLC + NOS-T* lines were generated by replacing the *COOLAIR* promoter with the *MAF2* terminator and *NOS* terminator, respectively (Li et al. 2018), while the *FLC_ΔCOOLAIR_* line was generated by deleting a 324-bp region in the *COOLAIR* promoter, downstream from *FLC* 3′ UTR, using the CRISPR method (Luo et al. 2019). The novel *CAS* transcripts originating from *FLC* intron 1 described above were detected in all three lines, with an even higher level in *FLC + MAF2-T* and *FLC + NOS-T* (Supplemental Fig. S5B–D). In addition, similar to *TEX1.0*, antisense transcript start sites were detected in the *MAF2-T* terminator in the *FLC + MAF2-T* line (Supplemental Fig. S5D). Similar findings of cryptic promoter usage after disruption of upstream promoters, and alternative transcripts arising when antisense transcription is perturbed, have been reported in *S. cerevisiae* (Mayer et al. 2015; Kim et al. 2016). The generation of novel *COOLAIR-CAS* transcripts from intragenic regions in *FLC* at each attempt to remove *COOLAIR*, including the CRISPR deletion of the endogenous *COOLAIR* promoter (*FLC_ΔCOOLAIR_*), suggests a tight interconnection between antisense transcription and chromatin state at the locus.

## Discussion

**Figure 5.**
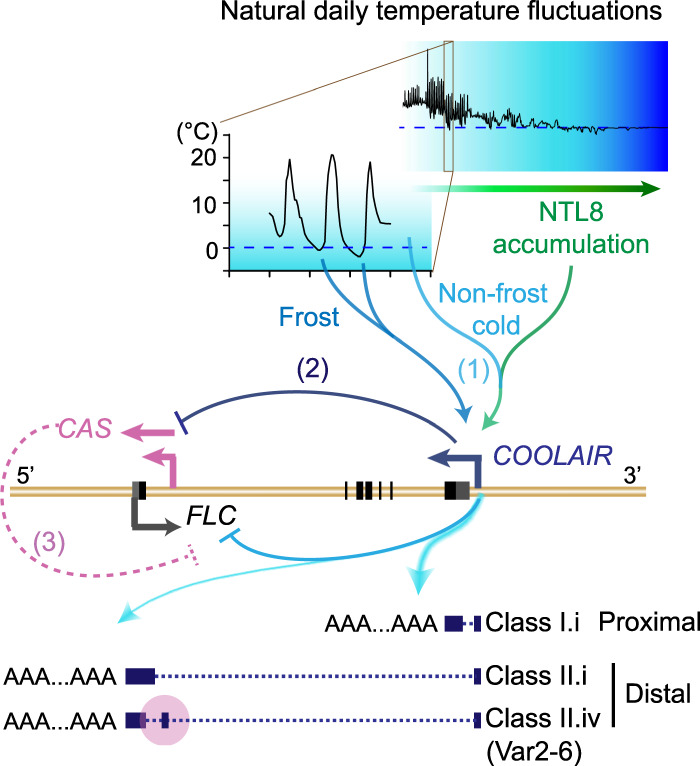
A schematic illustration of *COOLAIR* regulation of *FLC* expression. (1) Natural low temperatures (indicated by graded blue) promote NTL8 slow accumulation (graded green arrow), providing the long-term cold information for *COOLAIR* up-regulation and *FLC* regulation. Frosts function as strong cues to enhance *COOLAIR* up-regulation, conferring transcriptional plasticity to *FLC*. Furthermore, proximal polyadenylation of *COOLAIR* is enhanced by natural low temperatures, including freezing, supporting the mechanism tightly linking the altered 3′ processing/polyadenylation of *COOLAIR* to the transcriptional state at the *FLC* locus (Liu et al. 2010; Marquardt et al. 2014; Fang et al. 2020). (2) *CAS* transcription is inhibited by transcription from the upstream *COOLAIR* promoter, possibly influenced by the cold-promoted proximal polyadenylation of *COOLAIR*. (3) *CAS* originates from the region (shaded in pink) where the class II.iv exon is alternatively spliced (Li et al. 2015), highlighting its importance in the *COOLAIR* regulation of *FLC* transcription.

Plants effectively use fluctuating temperature cues to judge seasonal progression, but the molecular mechanisms underlying this are poorly understood. Through field studies of different *Arabidopsis* genotypes, we have shown how a temperature dip below freezing hyperinduces transcription of antisense transcripts at the floral repressor locus *FLC* to facilitate transcriptional silencing (Fig. 5). Since in natural field conditions, colder weather generally follows the first autumn frost, the hyperinduction of *COOLAIR* by freezing may be one of the many cues used by plants to monitor seasonal progression. The evolutionary significance of this remains to be explored. The ability to respond to acute or ambient cold temperature may provide the plasticity important for adaptation to different climates. This would then have parallels to environmentally regulated gene regulation in yeast, where antisense transcription has been shown to induce faster and higher amplitude changes in the associated gene in response to environmental cues (Xu et al. 2011; Beck et al. 2016; Cloutier et al. 2016). Subsequent frosts show weaker and variable effects on *COOLAIR* expression (Fig. 1; Supplemental Figs. S1, S2), likely due to the epigenetic silencing of the whole locus by the VIN3-dependent Polycomb switching mechanism that occurs after the cold-induced transcriptional silencing (Antoniou-Kourounioti et al. 2018; Hepworth et al. 2018).

How such small changes in temperature—from just above to below freezing—cause such a strong up-regulation of *COOLAIR* expression remains to be determined. An R loop generated by the invasion of *COOLAIR* into the DNA duplex at the *COOLAIR* promoter limits further rounds of antisense transcription (Sun et al. 2013). Whether freezing temperature alters the biophysical behavior of this structure to enable strong up-regulation of expression is an interesting possibility (Sun et al. 2007). NTL8 may also interface with the R-loop to influence *COOLAIR* expression. We have recently found that NTL8 is a direct regulator of *VIN3*, providing long-term cold information to *VIN3* through the mechanism of reduced dilution from slower growth (Zhao et al. 2020). It would seem likely that NTL8 provides long-term cold information to *COOLAIR* (Fig. 5)*,* but whether NTL8 or its homologs are also involved in the response of *COOLAIR* to acute freezing temperature remains to be addressed.

How *COOLAIR* transcription influences the transcriptional output of *FLC* remains to be resolved, but the ectopic transcription of *COOLAIR* in the *ntl8-D* mutant demonstrated causality between up-regulation of *COOLAIR* and *FLC* down-regulation at the same gene copy (Fig. 2). Antisense transcription has been shown to alter sense transcription dynamics in a chromatin-dependent manner in yeast and human cells (Murray et al. 2015; Brown et al. 2018). This appears to also be the case for *FLC* as removal of *COOLAIR* disrupted the synchronized replacement of H3K36me3 with H3K27me3 at the intragenic *FLC* nucleation site during the cold (Csorba et al. 2014). It is also possible that *COOLAIR* transcription reduces functionality of *trans* factors or gene/intronic loops that promote *FLC* transcription (Crevillén et al. 2013; Li et al. 2018).

The failed attempts with a range of transgenes to completely remove *COOLAIR* helps explain the confusion on *COOLAIR* function. All the independent transgenes analyzed produce new antisense transcripts initiating in the first intron. Consistent with this, recent genome-wide measurements of TSSs showed that extensive alternative intragenic transcriptional initiation occurs in *Arabidopsis*, and this can be affected by cold (Kindgren et al. 2018), cotranscriptional RNA degradation (Thieffry et al. 2020; Thomas et al. 2020), or mutants disrupted in chromatin signaling (Nielsen et al. 2019; Le et al. 2020). The activation of alternative TSSs significantly influences transcription from nearby TSSs, and thus is important for plant development and adaptation to environmental changes (Kindgren et al. 2018; Nielsen et al. 2019; Le et al. 2020; Thieffry et al. 2020; Thomas et al. 2020). Such *CAS* transcripts have been found to initiate globally from promoter-proximal exon–intron boundaries across the *Arabidopsis* genome and are correlated with promoter-proximal RNA Pol II stalling, a checkpoint for transcriptional regulation (Adelman and Lis 2012; Core and Adelman 2019; Kindgren et al. 2020). Interestingly, the major *COOLAIR CAS* transcripts originate in a region encompassing the alternative splice site of the distal polyadenylated *COOLAIR* class II.iv. This isoform has higher abundance in the natural accession Var2-6 and is associated with increased *FLC* expression through a cotranscriptional mechanism involving capping of the *FLC* nascent transcript (Fig. 5; Li et al. 2015). Moreover, the slower *FLC* shutdown rate in Var2-6 in the field supports the importance of this checkpoint in *FLC* transcription regulation (Hepworth et al. 2020). Similar to other systems (Lenstra et al. 2015), these *CAS* transcripts are likely suppressed at the endogenous locus by *COOLAIR* transcription from the upstream native promoter. This suppression may be influenced by the cold promotion of proximal 3′ processing/polyadenylation of *COOLAIR*. This would parallel *FLC* silencing in warm conditions, promoted by the alternative 3′ processing of *COOLAIR* (Liu et al. 2010; Marquardt et al. 2014; Fang et al. 2020). We envision that these *CAS* transcripts might be differentially expressed when *COOLAIR* transcription is altered, for example, during the initial freezing-dependent *COOLAIR* spike, possibly contributing to the switch of the local chromatin/transcription states. It will be informative in the future to capture the dynamics of these low abundant *CAS* transcripts during vernalization. Our current understanding of the many ways *COOLAIR* regulates *FLC* is shown in Figure 5.

Overall, our work reveals how first frost acts as a seasonal cue to up-regulate *COOLAIR* and transcriptionally repress *FLC*. Such a temperature-regulated antisense–sense circuitry endows a transcriptional plasticity to the *FLC* locus to effectively respond to the natural fluctuating temperatures of autumn. On a different timescale, but also cold-induced, the Polycomb nucleation mechanism then locks in the *FLC* silenced transcriptional state to maintain the epigenetic memory of cold exposure. Dissection of the vernalization response has thus elucidated the mechanisms used by plants to translate natural temperature fluctuations into long-term seasonal information.

## Materials and methods

No statistical methods were used to predetermine sample size. Field experiments were randomized in a complete-block design as described (Hepworth et al. 2020), with sample size chosen on the basis of feasibility and to buffer against sample loss. Laboratory experiments were not randomized and investigators were not blinded to allocation during experiments and outcome assessment. Sampling in all cases was performed by collecting material from new plants (not repeated sampling) for replicates and also between time points.

### Plant materials

All near-isogenic lines in this study were previously described in Hepworth et al. (2020). *FLC::FLC-TEX1.0* and *FLC::FLC15* WT707 were previously described in Csorba et al. (2014). *FLC + MAF2-T*, *FLC + NOS-T*, and *FLC_ΔCOOLAIR_* were generated as previously described (Li et al. 2018; Luo et al. 2019). The other two *ntl8-D* alleles (*ntl8-D1* and *ntl8-D2*), GFP-NTL8-D2 transgenic line, and NTL8 overexpression lines (*ntl8-OE1* [Salk_866741], *ntl8-OE2* [Salk_587226], and *ntl8-OE3* [*35S::HA-NTL8* transgenic line]) were previously described (Zhao et al. 2020).

The *COOLAIRprom::luciferase* reporter line was generated by transforming the *COOLAIRprom-luciferase* construct, as previously described (Sun et al. 2013), into Col *FRI* plants. A transgenic line containing a single-copy transgene was selected as the progenitor line (named as CTL) for the forward genetic screen.

*FLC::FLC-TEX2.0* was generated by inserting a NOS terminator fragment within the first exon of *COOLAIR* within a ∼12-kb genomic fragment, using adjacent EcoRI and SalI restriction sites immediately downstream from the *FLC* sense 3′ UTR and the primers “fragment-1-F-1653” (5′*-*GCTTAACGAGCTTGCACACA-3′) with “fragment-1-R-EcoRI-1653” (5′-GAGGAATTcaagatctcgatgcaattctcac-3′) and “fragment-2-F-SalI” (5′-AGAGTCGACagtgtatgtgttcttcacttctgtcaa-3′) with “fragment-2-R” (5′-TATGGAAGAGGTCGGTCACG-3′). The assembled fragment was cloned into pCambia1300, which was transformed into the *Arabidopsis flc-2 FRI* genotype with a floral dipping method. Transformants were selected on medium supplemented with hygromycin (Sigma-Aldrich H0654), and single-copy transformants were selfed to generate homozygous T3 lines. Both *FLC* and *COOLAIR* expressions were screened before vernalization and after a 2-wk vernalization treatment to identify a single representative line, *TEX2.0-472*.

### Plant growth conditions

Plant were generally grown in growth conditions as described previously (Berry et al. 2015; Rosa et al. 2016; Antoniou-Kourounioti et al. 2018; Hepworth et al. 2018, 2020; Zhao et al. 2020).

#### Field experiments

Details for field experiments have been described previously (for 2016–2017 winter, see Antoniou-Kourounioti et al. 2018; for 2014–2015 winter, see Hepworth et al. 2018; for both 2016–2017 and 2014–2015 winters, see Hepworth et al. 2020). Briefly, the experiment site in the north was at Ramsta (62°50.988′ N, 18°11.570′ E), and in the south was at Ullstorp (56°06.6721′ N, 13°94.4655′ E). Plants were sown and moved to the field site as follows: Norwich, sown into position on September 29, 2014; South Sweden, sown on September 24, 2014, and moved on October 8, 2014; North Sweden—first year, early planting sown on August 26, 2014, and moved on September 11, 2014, and late planting sown on September 8, 2014, and moved on September 24, 2014; and North Sweden—second year, sown on August 12, 2016, and moved on August 24, 2016.

#### Laboratory experiments

In general, seeds were stratified after sowing for 3 d at ∼4°C–5°C. Briefly, for RNA analysis and ChIP experiments, plants were grown on Murashige and Skoog (MS) agar plates without glucose. For microscopy, plants were grown on MS plates with 1% agar placed vertically. For nonvernalized (NV) conditions, plants were grown for 10–12 d at long photoperiod conditions (16-h light, 8-h dark with constant 20°C), while for vernalization treatment, plants were moved to 5°C cold treatment at short photoperiod conditions (8-h light, 16-h dark with constant 5°C) after growing at long photoperiod conditions for 7 d if not specified.

#### Response of TEX1.0 and TEX2.0 lines in laboratory conditions (Fig. 3E–G)

Seeds were sown on plates with selective antibiotic and MS agar media without glucose. Plants were grown for 10 d postgermination in long days at 20°C and then grown for an additional 3 d before NV sampling or moved to a growth room for vernalization with 8-h light and grown for 2 or 4 wk (2WV or 4WV) at 5°C, before transferring seedlings to soil and growing for 20 d at 22°C/20°C with 16-h light/8-h darkness (4WT20). Three replicates of >15 seedlings (NV, 2WV, and 4WV) or three replicates of three pooled leaf/meristem tissue (4WT20) were screened for expression.

Flowering time was analyzed as previously described (Liu et al. 2010). Briefly, plants were vernalized for 4 wk at 5°C with 8-h light before being transferred to soil at 22°C/20°C with 16-h light/8-h darkness. The days plants took to bolting when flower buds were visible at the shoot apical meristem were counted as a measurement of flowering time. Counting was stopped after 50 d as all plants had flowered for controls and *TEX1.0* and nothing for *TEX2.0*.

#### Recreation of field freezing conditions in the laboratory (Supplemental Fig. S3C–E)

Plants were grown in a nonvernalizing growth chamber in long days for 1 wk at 22°C/20°C 16-h light/8-h darkness and then transferred to a growth chamber (Conviron) with the temperatures and light period matching the week before the first frost (to allow acclimation) and the day following the morning frost. Temperatures and light times are shown in Supplemental Table S2. Three biological replicates of more than five seedlings were sampled at 17:00 and 20:00 on the seventh day, the night before freezing; at 07:00, 09:00, 11:00, 13:00, 15:00, 17:00, and 20:00 on the eighth day, when freezing occurred at 10:00 and 13:00–14:00; and at 20:00 on the following day.

#### Fluctuating freezing experiments (Figs. 1B–E, 3H,I; Supplemental Fig. S3F–J)

Col *FRI* seeds were sown onto soil. Nonvernalized plants were grown for 10 d in the nonvernalizing growth chamber and sampled at six time points over 24 h before plants were then moved to 8-h light/16-h darkness growth chambers for vernalization Light was on from 09:00 to 17:00. For vernalization, plants were treated with three different temperature regimes: constant 5°C, daily fluctuating 5°C (3°C–9°C) and daily fluctuating 5°C (−1°C to 12°C) (Fig. 1B). Three biological replicate samples were taken at six time points over 24 h after 2-wk exposure to vernalization. For the NV samples, plants were sampled at 12:00, 16:00, 20:00, and 24:00 and at 08:00 twice 24 h apart. For the vernalization samples, plants were sampled at 12:00, 16:00, 20:00, and 24:00 and at 09:00 twice 24 h apart. Lights came on at midnight for NV and at 09:00 in vernalization treatments. For Figure 3, H and I, experiments were performed as described above, except sampling was performed at the time point (09:00). For Supplemental Figure S3, F–J, experiments were performed following the above procedures, except that Col *FRI* seeds were sown onto medium in Petri dishes and were sampled at the time point (09:00).

### Mutagenesis, genetic screening, and gene cloning of *ntl8-D3* mutation

Mutagenesis was carried out following the procedures previously described (Liu et al. 2010). Around 20 M2 (mutagenesis generation 2) seeds from each single M1 plant were screened by being sown on MS medium and stratified for 3 d in the cold (5°C). After growing in a growth cabinet for 10 d, the M2 seedlings were assayed for the bioluminescence with 1 μM luciferin (Promega E1603) under a CCD camera (NightOwl). A mutant was identified to also show high *VIN3* expression in warm conditions (Supplemental Fig. S4N), similar to what we found in *ntl8-D1* and *ntl8-D2* mutants (Zhao et al. 2020). Further Sanger sequencing showed that the mutant carries a mutation in *AT2G27300* and so was named *ntl8-D3* (Supplemental Fig. S4G,O).

### RNA extraction and QPCR

Total RNA was extracted as previously described (Box et al. 2011). Genomic DNA was digested with TURBO DNA-free (Ambion Turbo DNase kit AM1907) according to the manufacturer's guidelines, before reverse transcription was performed. The reverse transcription was performed with the SuperScript III reverse transcriptase (ThermoFisher 18080093) following the manufacturer's protocol using gene-specific primers. Relevant primers are listed in Supplemental Tables S3 and S4. For field experiments, RNA extraction and QPCR were performed as described (Antoniou-Kourounioti et al. 2018; Hepworth et al. 2018, 2020). In brief, analysis of qPCR results was performed with LinReg with normalization to the geometric means of the *At5g25760* (“*PP2A*”) and *At1g13320* (“*UBC*”) control genes. The same analysis was also used in Figure 1, C–E, and Supplemental Figure S3, C–E. The rest results were normalized to single reference gene, *UBC*. Primers used are described in Supplemental Table S3.

### Microscopy

For detecting the fluorescence of FLC-Venus, confocal imaging was performed using a 20×/0.7 NA multi-immersion lens, with water as the immersion fluid on a Leica TCS SP8 X confocal microscope following the procedures in Berry et al. (2015). Roots were immersed in 2 μg/mL propidium iodide (Sigma-Aldrich P4864) to label the cell wall. FLC-Venus was excited with illumination at 514 nm (Argon ion laser). Emissions from Venus were detected between 518 nm and 555 nm using a cooled Leica HyD SMD detector in photon-counting mode. Propidium iodide was detected simultaneously with FLC-Venus by collecting emissions between wavelengths 610 nm and 680 nm. To allow comparison between treatments, the same settings were used for images in both the wild type and *ntl8-D3* mutant.

The smRNA FISH experiment was performed following the procedures described previously (Rosa et al. 2016).

### Chromatin immunoprecipitation (ChIP)

NTL8 protein ChIP experiments were carried out following the methods described in Zhao et al. (2020). Briefly, nuclei were extracted from 3 g of materials with 30 mL of Honda buffer (0.4 M sucrose, 2.5% Ficoll, 5% dextran T40, 25 mM Tris-HCl at pH 7.4, 10 mM MgCl_2_, 0.5% Triton X-100, 0.5 mM PMSF, proteinase inhibitor cocktail [Roche 04693159001], 5 mM DTT). After nuclei extraction, purified nuclei were lysed by RIPA buffer (1× PBS, 1% Igepal CA-630 [Sigma I8896], 0.5% sodium deoxycholate, 0.1% SDS, proteinase inhibitor cocktail) and then fragmented by sonication (Diagenode Bioruptor). After sonication, the fragmented chromatin extract was cleared by centrifugation at 13,000 rpm for 15 min at 4°C before immunoprecipitation. The immunoprecipitations were performed with GFP-trap beads (Chromotek GTMA-20) for GFP-NTL8-D2 (Fig. 2G) and anti-HA magnetic beads (Pierce 88836) for HA-NTL8 (Supplemental Fig. S4H) in the *ntl8-OE3* line. Relevant primers are listed in Supplemental Table S3.

### 5′ RACE

Five micrograms of total RNA was first treated with calf intestine alkaline phosphatase (CIAP; Merk Sigma P4978) for 1 h at 37°C and purified with phenol:chloroform. After 5′ cap removing for 1 h at 37°C with cap-clip acid pyrophosphatase (CCAP; Cambio C-CC15011H), 1 μL of 5′ RACE adapter 0.3 μg/μL; (5′-GCUGAUGGCGAUGAAUGAACACUGCGUUUGCUGGCUUUGAUGAAA-3′) was ligated to a half volume of the CIAP/CCAP-treated RNA (2.5 μL) by T4 RNA ligase (NEB M0204S) for 2 h at 37°C. The CIAP-treated RNA without following CCAP treatment was used as control. All the 5′ RACE adapter-ligated RNA was then reverse transcribed by SuperScript III reverse transcriptase (ThermoFisher 18080093) with a distal *COOLAIR*-specific primer, RT-R, with *TUB6* as a control (Fig. 4B). The cDNA was submitted to nested PCR, and the subsequently purified PCR products were ligated to a T-vector (Supplemental Fig. S5A). At least 48 colonies from each RNA sample were examined by PCR before at least two colonies from each different sized PCR product were sent to Sanger sequencing. Oligos for 5′ RACE are listed in Supplemental Table S4.

## Supplementary Material

Supplemental Material
