# Supplementary material for: Natural temperature fluctuations promote COOLAIR regulation of FLC
Source: Genes Dev. 2021 Jun;35(11-12):888–98. doi: 10.1101/gad.348362.121 (PMC8168555; doi:10.1101/gad.348362.121)
Supplement: Supplemental Material [file supp_35_11-12_888__DC1.html]

Natural temperature fluctuations promote COOLAIR regulation of FLC — Supplemental Material 

# Natural temperature fluctuations promote *COOLAIR* regulation of *FLC*

## Supplemental Material

- Supplemental\_Data.pdf
