# Supplementary material for: Natural temperature fluctuations promote COOLAIR regulation of FLC
Source: Genes Dev. 2021 Jun;35(11-12):888–98. doi: 10.1101/gad.348362.121 (PMC8168555; doi:10.1101/gad.348362.121)
Supplement: Supplemental Material [file supp_gad.348362.121_Supplemental_Data.pdf]

Supplemental Figure S1-5, and Table S1-4

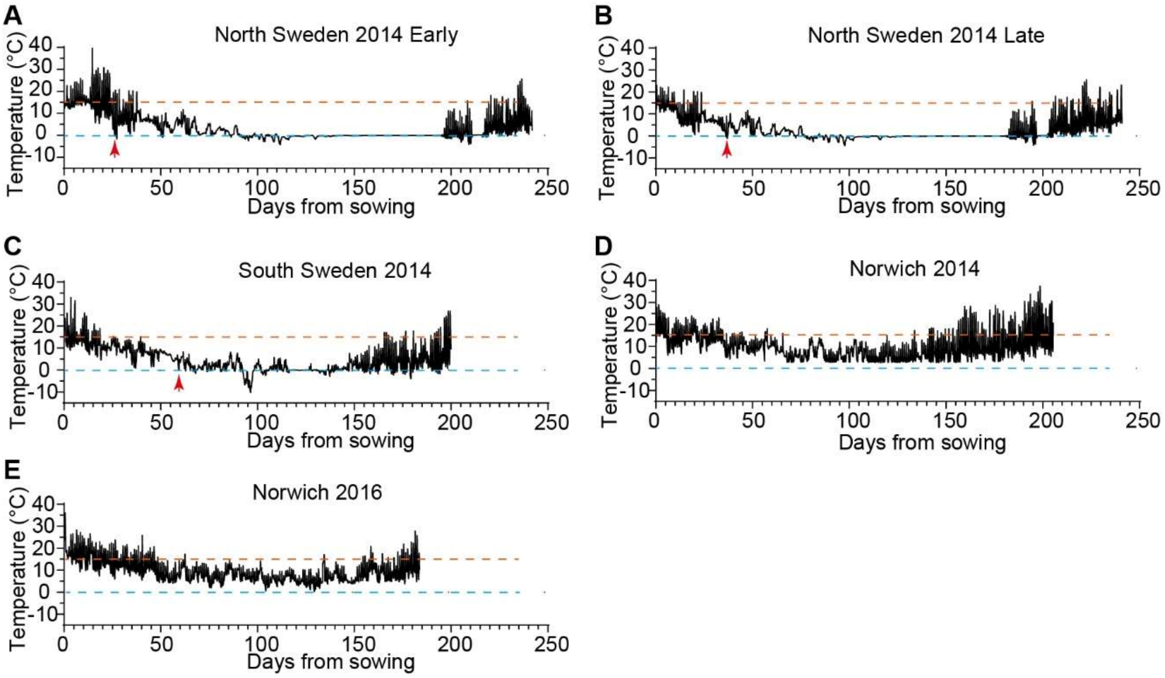

Supplemental Figure S1. Temperatures experienced by plants in the field (Antoniou-Kourounioti et al. 2018; Hepworth et al. 2018; Hepworth et al. 2020**).**

(A) Temperature profile in the early sowing of first year (2014) in North Sweden. *(B)* Temperature profile in the late sowing of first year (2014) in North Sweden. *(C)* Temperature profile in the sowing of first year (2014) in South Sweden. *(D)* Temperature profile in the sowing of first year (2014) in Norwich. *(E)* Temperature profile in the sowing of second year (2016) in Norwich. Red arrows indicate the first frost. Orange lines indicate 15°C, and light blue dashed lines indicate 0°C.

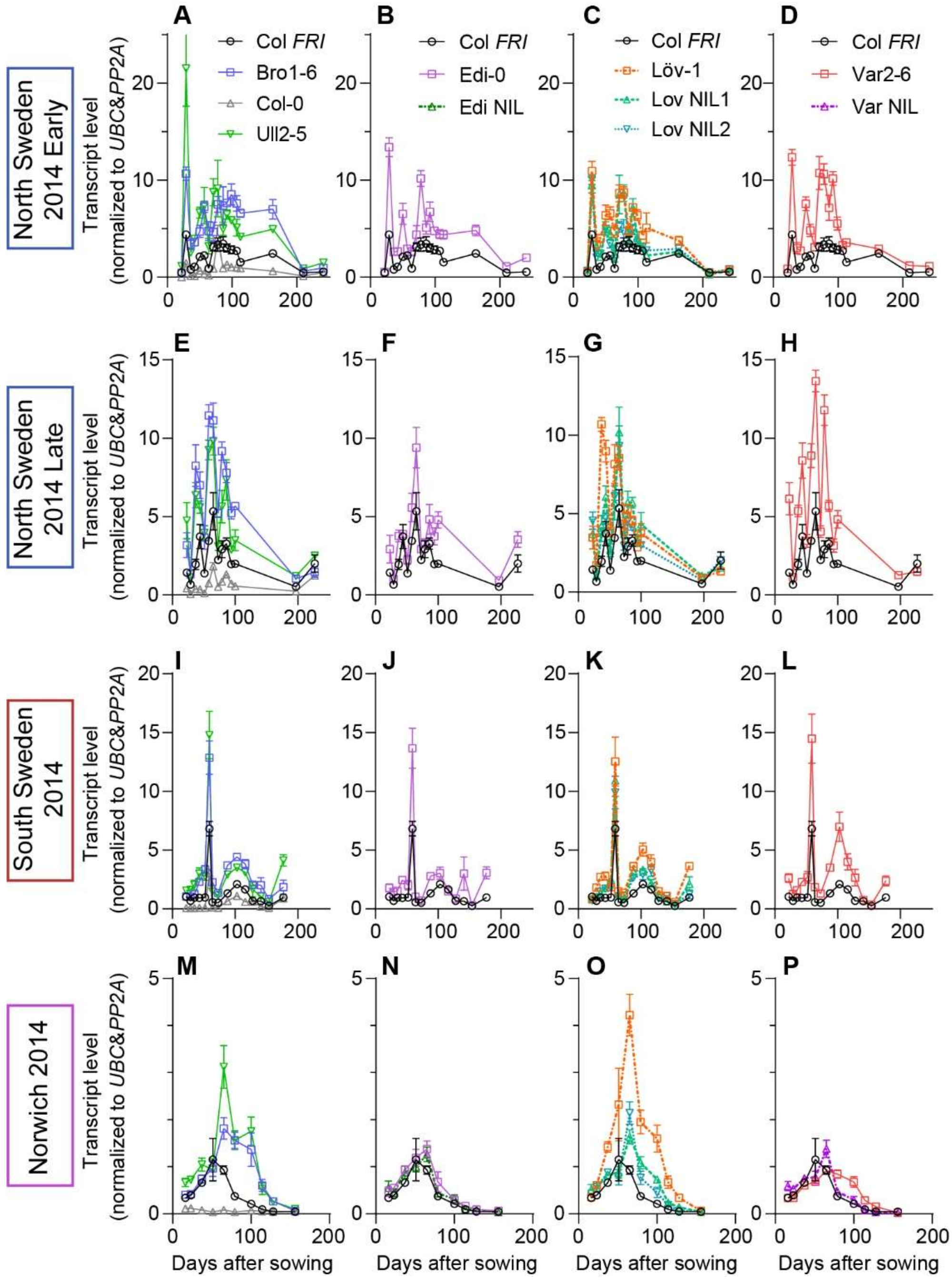

Supplemental Figure S2. *COOLAIR* expression in the field across all three sites in 2014-5.

COOLAIR expression data, normalised to the control sample for 2014-5 (see Methods). Error bars show s.e.m. Experiment for the two genotypes of Edi NIL and Var NIL were performed only in ‘Norwich 2014’ in the bottom panel.

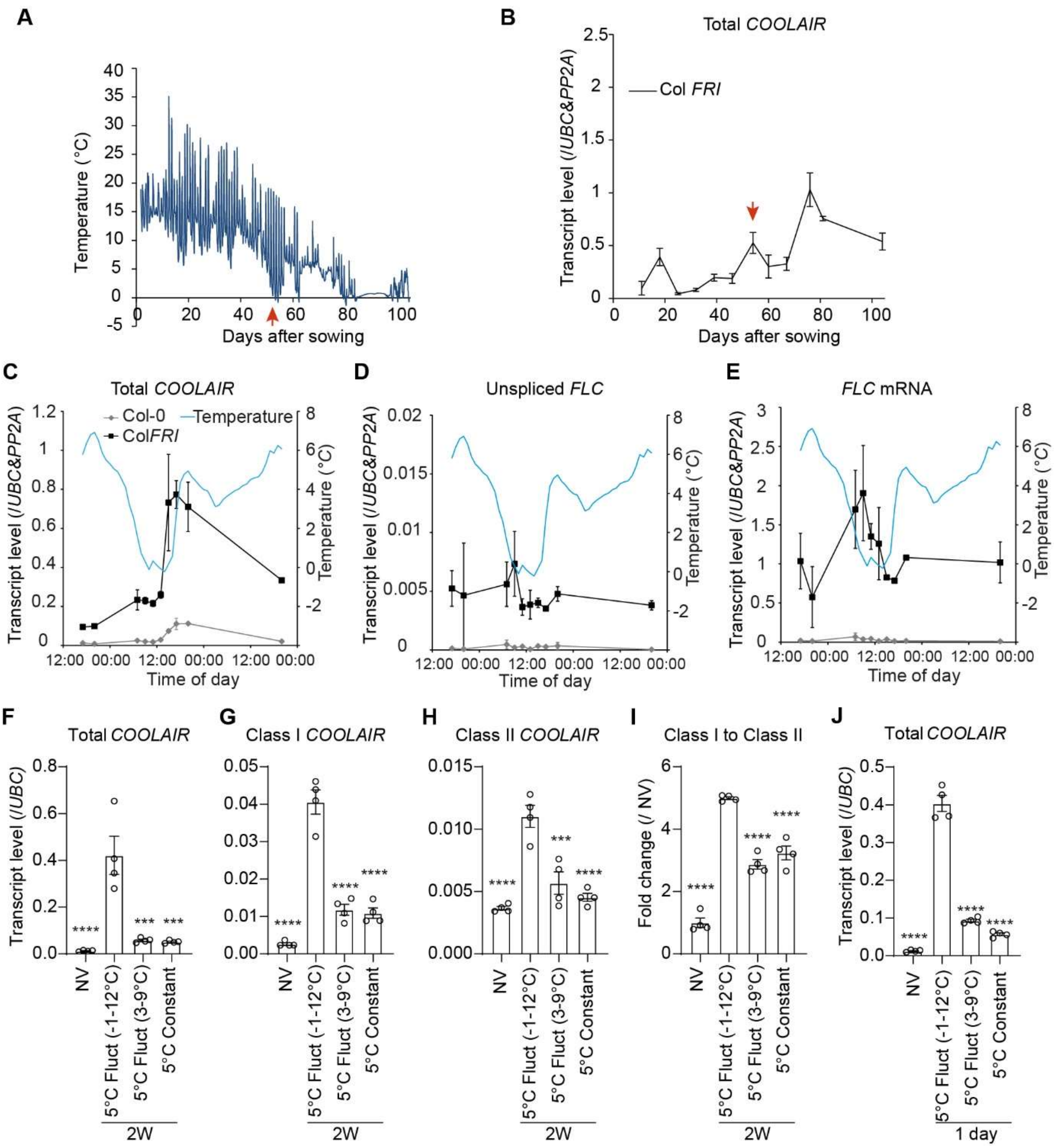

Supplemental Figure S3. ***COOLAIR*** **expression peaks in response to freezing both in the field and the laboratory.**

(A) Hourly temperature readings from plant-level in North Sweden 2016. *(B) COOLAIR* levels in the Col *FRI* over autumn, responding both to prolonged cold and, more rapidly, to freezing (e.g. 5th October, 54 days, indicated by an arrow), (see Methods). Error bars show s.e.m. *(C-E)* Expression of *COOLAIR (C)*, upspliced *FLC* transcript level *(D)*, and *FLC* mRNA *(E)* in Col *FRI* and Col-0 over the day of the first frost in plants grown for two weeks

at 20°C, then kept for a week in a growth cabinet set to recreate the temperature conditions and approximate photoperiod of the week of the first frost in South Sweden in 2014 (in Fig.1A). Error bars show s.e.m. *(F-I)* qPCR total *COOLAIR (F)*, proximal Class I *COOLAIR (G)*, and distal class II *COOLAIR (H)* transcript levels before and after 2 weeks in the conditions shown in Fig.1B. Levels were normalized to *UBC*. *(I)* Ratio of proximal to distal *COOLAIR* with the data from *(F-H)*, normalized to NV. *(J)* qPCR total *COOLAIR* transcript levels before and after 1 day in the conditions shown in Fig.1B. Levels were normalized to *UBC*. Error bars show s.e.m. of four biological replicates. One way analysis of variance (ANOVA) with Dunnett's multiple comparisons test was performed, and significances for individual comparisons of interest are shown. *** $P<0.001$, **** $P<0.0001$.

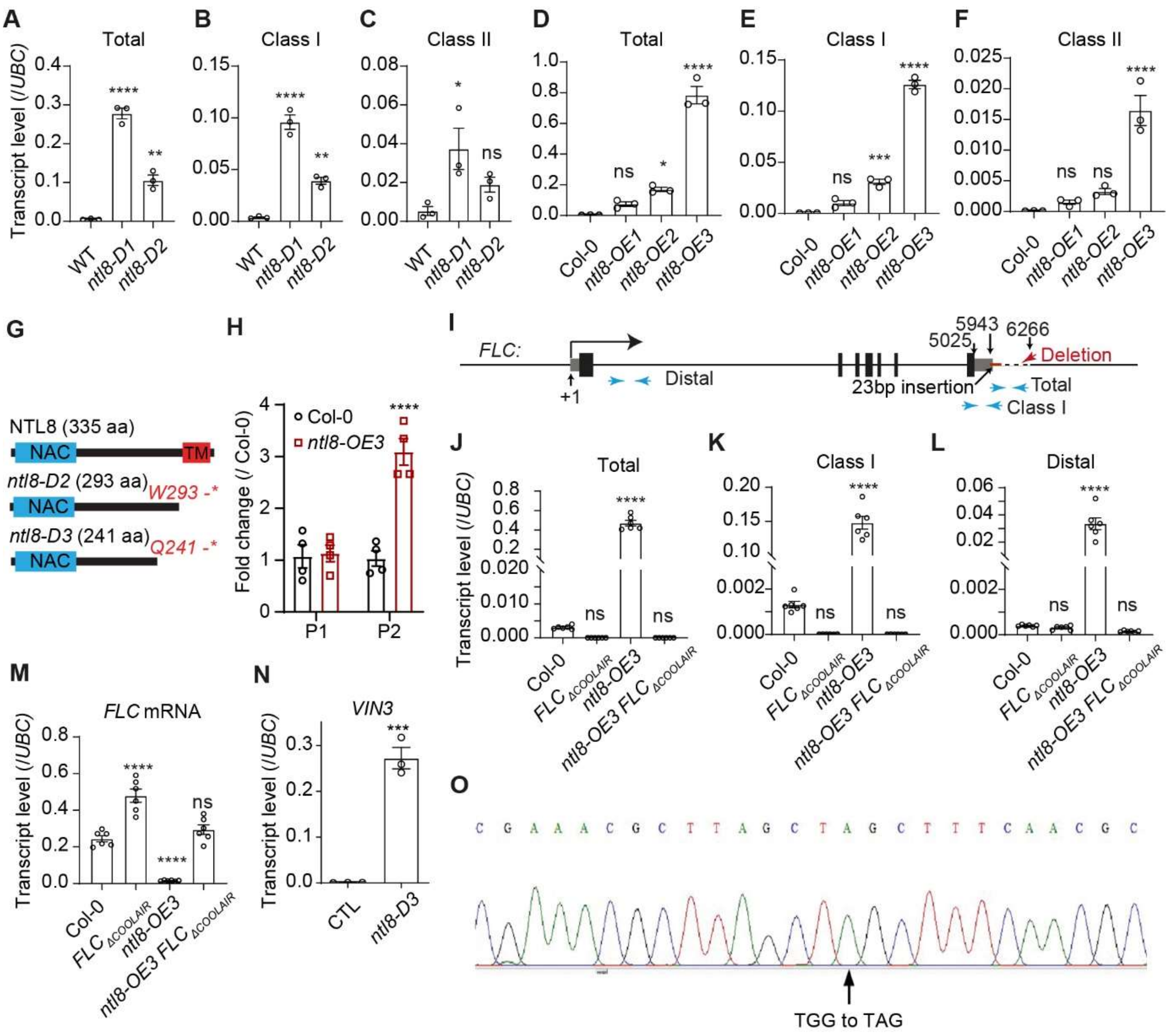

Supplemental Figure S4. *COOLAIR* is upregulated in *ntl8-D* and *ntl8-OE* mutants in the warm.

(A-C) qPCR Total *COOLAIR (A)*, Class I *COOLAIR (B)*, Class II *COOLAIR (C)* transcript levels in warm (20°C) in WT, *ntl8-D1* and *ntl8-D2* mutants. WT indicates the progenitor line of *ntl8-D1* and *ntl8-D2* mutants. *(D-F)* qPCR Total *COOLAIR (D)*, Class I *COOLAIR (E)*, Class II *COOLAIR (F)* transcript levels in warm (20°C) in Col-0, *ntl8-OE1, ntl8-OE2* and *ntl8-OE3* mutants. Levels normalized to *UBC*. Error bars show s.e.m. of three biological replicates. *(G)* Schematic of the predicted proteins with the *ntl8-D2* and *ntl8-D3* mutations. Light blue box indicates NAC (NAM/ATAF/CUC) domain, red box indicates transmembrane domain (TM). W indicates Tryptophan, Q indicates Glutamine, * indicates stop codon. *(H)*

ChIP analysis of NTL8 binding at *COOLAIR* region with HA- tagged NTL8 in the NTL8 overexpression line *ntl8-OE3*. Control: Col-0. Positions of primer P1 and P2 are indicated on the diagram in Fig.2G. Error bars show s.d. of four replicates. *(I)* diagram of the $FLC_{\Delta COOLAIR}$ and relative binding sites of Total, proximal polyadenylated Class I and distal polyadenylated Class II primers. *(J-M)* qPCR analysis of Total *(J)*, Class I *(K)*, Class II *(L) COOLAIR* and *FLC* mRNA *(M)* transcript levels in warm (20°C) in Col-0, $FLC_{\Delta COOLAIR}$, *ntl8-OE3* and *ntl8-OE3* $FLC_{\Delta COOLAIR}$ mutants. Levels were normalized to *UBC*. Error bars show s.e.m. of six biological replicates. *(N)* qPCR *VIN3* transcript leve in warm (20°C) in CTL and *ntl8-D3* mutant. Levels normalized to *UBC*. Error bars show s.e.m. of three biological replicates. Two way analysis of variance (ANOVA) with Turkey's multiple comparisions test was performed for *(H)*, and One way analysis of variance (ANOVA) with Dunnett's multiple comparisons test was performed for the rest panels. Significances for individual comparisons of interest are shown. * $P<0.05$, ** $P<0.01$, *** $P<0.001$, **** $P<0.0001$. ns, no significance. *(O)* Sequencing chromatograms show a G to A mutation in the *At2g27300* gene sequence (indicated by the arrow).

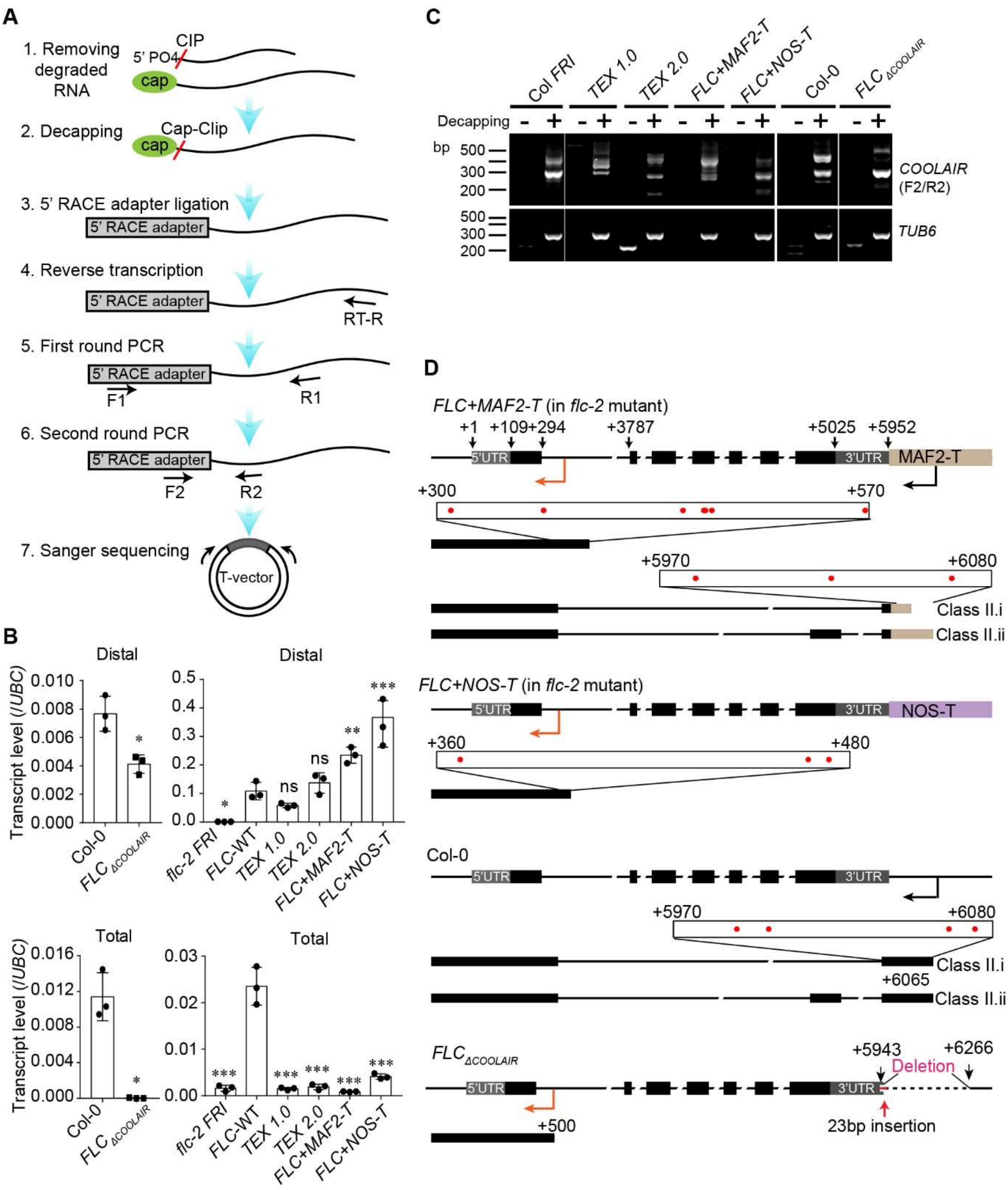

Supplemental Figure S5. Removal of *COOLAIR* promotes intragenic convergent antisense transcripts in other mutants.

(A) The procedure of 5' RACE (see Methods). *(B)* Expression level of antisense transcripts in NV (20°C) in wild type and *COOLAIR* mutants. *flc-2 FRI* was used as negative control. Error bars show s.e.m. for 3 biological replicates. * $P<0.05$, ** $P<0.01$, *** $P<0.001$ (Unpaired

two-tailed t test for left panel and Dunnett's multiple comparisons test for right panel). Primers used is illustrated in Fig. 3A. *(C)* Agarose gel showing the antisense transcripts detected by 5' RACE in the indicated NV (20°C) plants. *TUB6* was used as control. Samples without (-) or with (+) decapping treatment were indicated. Primers used were mapped in Fig.4B. *(D)* Schematic illustrations showing the antisense transcriptional start sites (TSS) mapped by 5' RACE in NV (20°C) plants. Untranslated region (UTR) of *FLC* is indicated by gray box and exons are represented by black boxes. Black arrows show the positions of *COOLAIR* TSS and orange arrows show those of the *CAS*. Antisense TSSs were mapped in the scaled boxes with red dots. Numbers indicate distance (bp) from *FLC* TSS. 23 bp insertion indicated by the red arrow was accidentally made during the CRISPR.

Supplemental Table S1. Summary of sequencing results of 5' RACE for distal antisense transcription at *FLC* locus in wild type and *COOLAIR* manipulating lines.

Col *FRI*			
Treatment	Position	Distance from *FLC* TSS	Clone number
NV	*FLC* exon1	189	2
	Class II.i	6005	1
	Class II.i	6065	1
	Class II.i	6067	4
	Class II.i	6072	1
	Class II.i	6076	4
	Class II.i	6077	4
	Class II.i	6078	1
	Class II.ii	5992	2
	Class II.ii	6041	5
	Class II.ii	6054	1
Treatment	Position	Distance from *FLC* TSS	Clone number
2WV	Class II.i	6036	8
	Class II.i	6061	2
	Class II.i	6067	2
	Class II.i	6076	2
	Class II.i	6093	1
	Class II.i	6097	3
	Class II.ii	5975	1
	Class II.ii	5992	2
	Class II.ii	6005	1
	Class II.ii	6014	1
	Class II.ii	6029	3
	Class II.ii	6040	2
	Class II.ii	6041	2
	Class II.ii	6070	1
	Class II.ii	6074	7
	Class II.ii	6076	2
TEX 1.0			
Treatment	Position	Distance from *FLC* TSS	Clone number
NV	*FLC* intron 1	492	3
	Class II.ii	with 76 bp RBCS sequence	2
Treatment	Position	Distance from *FLC* TSS	Clone number

2WV	Class II.ii	with 25 bp RBCS sequence	2
	Class II.ii	with 83 bp RBCS sequence	2
TEX 2.0			
Treatment	Position	Distance from *FLC* TSS	Clone number
NV	*FLC* intron 1	468	6
	FLC intron 1	691	1
Treatment	Position	Distance from *FLC* TSS	Clone number
2WV	*FLC* exon1	189	1
	FLC intron 1	500	2
	FLC intron 1	531	1
	FLC intron 1	672	4
	FLC intron 1	708	3
FLC+MAF2-T			
Treatment	Position	Distance from *FLC* TSS	Clone number
NV	Class II.i	with 30 bp MAF2 sequence	2
	Class II.i	with 75 bp MAF2 sequence	2
	Class II.ii	with 115 bp MAF2 sequence	3
	FLC intron 1	309	1
	FLC intron 1	367	2
	FLC intron 1	454	1
	FLC intron 1	467	1
	FLC intron 1	468	3
	FLC intron 1	472	1
	FLC intron 1	568	1
FLC+NOS-T			
Treatment	Position	Distance from *FLC* TSS	Clone number
NV	*FLC* intron 1	367	1
	FLC intron 1	468	3
	FLC intron 1	474	2
Col-0			
Treatment	Position	Distance from *FLC* TSS	Clone number
NV	Class II.i	5992	1
	Class II.i	6003	5
	Class II.i	6065	1
	Class II.i	6074	1

	Class II.ii	6065	2
$\mathit{FLC}_{\mathit{\Delta COOLAIR}}$			
Treatment	Position	Distance from *FLC* TSS	Clone number
NV	*FLC* intron 1	500	9

Supplemental Table S2. Temperatures and light times in the re-creation of field freezing conditions in the laboratory.

Day	Time	Temperatures	Light ON/OFF
01/06/2016	0h	8.05	
01/06/2016	1h	7.91	
01/06/2016	2h	7.79	
01/06/2016	3h	7.69	
01/06/2016	4h	7.58	
01/06/2016	5h	7.54	
01/06/2016	6h	7.55	
01/06/2016	7h	7.55	
01/06/2016	8h	7.53	
01/06/2016	9h	7.52	
01/06/2016	10h	7.53	
01/06/2016	11h	7.56	
01/06/2016	12h	7.65	
01/06/2016	13h	7.75	
01/06/2016	14h	7.86	ON
01/06/2016	15h	8.12	ON
01/06/2016	16h	8.36	ON
01/06/2016	17h	8.44	ON
01/06/2016	18h	8.48	ON
01/06/2016	19h	8.56	ON
01/06/2016	20h	8.57	ON
01/06/2016	21h	8.52	ON
01/06/2016	22h	8.49	ON
01/06/2016	23h	8.49	
02/06/2016	0h	8.53	
02/06/2016	1h	8.53	
02/06/2016	2h	8.49	
02/06/2016	3h	8.46	
02/06/2016	4h	8.33	
02/06/2016	5h	8.06	
02/06/2016	6h	7.89	
02/06/2016	7h	7.83	
02/06/2016	8h	7.76	
02/06/2016	9h	7.63	
02/06/2016	10h	7.53	
02/06/2016	11h	7.6	
02/06/2016	12h	7.35	
02/06/2016	13h	7.28	
02/06/2016	14h	7.28	ON
02/06/2016	15h	7.3	ON

02/06/2016	16h	7.54	ON
02/06/2016	17h	7.77	ON
02/06/2016	18h	7.94	ON
02/06/2016	19h	7.81	ON
02/06/2016	20h	7.62	ON
02/06/2016	21h	7.41	ON
02/06/2016	22h	7.25	ON
02/06/2016	23h	7.12	
03/06/2016	0h	7.11	
03/06/2016	1h	7.11	
03/06/2016	2h	7.06	
03/06/2016	3h	7.18	
03/06/2016	4h	7.3	
03/06/2016	5h	7.38	
03/06/2016	6h	7.37	
03/06/2016	7h	7.36	
03/06/2016	8h	7.27	
03/06/2016	9h	7.15	
03/06/2016	10h	6.89	
03/06/2016	11h	6.7	
03/06/2016	12h	6.65	
03/06/2016	13h	6.9	
03/06/2016	14h	6.96	ON
03/06/2016	15h	6.99	ON
03/06/2016	16h	7	ON
03/06/2016	17h	7.06	ON
03/06/2016	18h	7.1	ON
03/06/2016	19h	7.07	ON
03/06/2016	20h	6.94	ON
03/06/2016	21h	6.8	ON
03/06/2016	22h	6.67	ON
03/06/2016	23h	6.67	
04/06/2016	0h	6.76	
04/06/2016	1h	6.79	
04/06/2016	2h	6.79	
04/06/2016	3h	6.82	
04/06/2016	4h	6.84	
04/06/2016	5h	6.88	
04/06/2016	6h	6.84	
04/06/2016	7h	6.85	
04/06/2016	8h	6.78	
04/06/2016	9h	6.78	
04/06/2016	10h	6.82	
04/06/2016	11h	6.84	
04/06/2016	12h	6.75	

04/06/2016	13h	6.71	
04/06/2016	14h	6.65	ON
04/06/2016	15h	6.7	ON
04/06/2016	16h	6.83	ON
04/06/2016	17h	6.99	ON
04/06/2016	18h	7.16	ON
04/06/2016	19h	7.32	ON
04/06/2016	20h	7.44	ON
04/06/2016	21h	7.25	ON
04/06/2016	22h	7.15	ON
04/06/2016	23h	7.15	
05/06/2016	0h	7.11	
05/06/2016	1h	7.09	
05/06/2016	2h	7.04	
05/06/2016	3h	7.01	
05/06/2016	4h	7	
05/06/2016	5h	6.97	
05/06/2016	6h	6.91	
05/06/2016	7h	6.84	
05/06/2016	8h	6.76	
05/06/2016	9h	6.76	
05/06/2016	10h	6.68	
05/06/2016	11h	6.68	
05/06/2016	12h	6.63	
05/06/2016	13h	6.48	
05/06/2016	14h	6.37	ON
05/06/2016	15h	6.17	ON
05/06/2016	16h	6	ON
05/06/2016	17h	5.88	ON
05/06/2016	18h	5.69	ON
05/06/2016	19h	5.56	ON
05/06/2016	20h	5.39	ON
05/06/2016	21h	5.22	ON
05/06/2016	22h	5.09	ON
05/06/2016	23h	4.98	
06/06/2016	0h	4.96	
06/06/2016	1h	4.95	
06/06/2016	2h	5	
06/06/2016	3h	5	
06/06/2016	4h	4.95	
06/06/2016	5h	4.85	
06/06/2016	6h	4.71	
06/06/2016	7h	4.59	
06/06/2016	8h	4.47	
06/06/2016	9h	4.35	

06/06/2016	10h	4.26	
06/06/2016	11h	4.14	
06/06/2016	12h	4.06	
06/06/2016	13h	4.03	
06/06/2016	14h	4.05	
06/06/2016	15h	4.08	ON
06/06/2016	16h	4.18	ON
06/06/2016	17h	4.27	ON
06/06/2016	18h	4.33	ON
06/06/2016	19h	4.41	ON
06/06/2016	20h	4.3	ON
06/06/2016	21h	4.1	ON
06/06/2016	22h	3.92	ON
06/06/2016	23h	3.82	
07/06/2016	0h	3.79	
07/06/2016	1h	3.78	
07/06/2016	2h	3.85	
07/06/2016	3h	3.87	
07/06/2016	4h	3.9	
07/06/2016	5h	3.95	
07/06/2016	6h	4.03	
07/06/2016	7h	4.09	
07/06/2016	8h	4.18	
07/06/2016	9h	4.24	
07/06/2016	10h	4.29	
07/06/2016	11h	4.3	
07/06/2016	12h	4.31	
07/06/2016	13h	4.35	
07/06/2016	14h	4.41	
07/06/2016	15h	4.56	ON
07/06/2016	16h	4.9	ON
07/06/2016	17h	5.37	ON
07/06/2016	18h	5.54	ON
07/06/2016	19h	5.52	ON
07/06/2016	20h	5.58	ON
07/06/2016	21h	5.51	ON
07/06/2016	22h	5.37	ON
07/06/2016	23h	5.3	
08/06/2016	0h	5.27	
08/06/2016	1h	5.22	
08/06/2016	2h	5.19	
08/06/2016	3h	5.07	
08/06/2016	4h	5.09	
08/06/2016	5h	5.02	
08/06/2016	6h	5.02	

08/06/2016	7h	4.99	
08/06/2016	8h	4.97	
08/06/2016	9h	4.92	
08/06/2016	10h	4.88	
08/06/2016	11h	4.77	
08/06/2016	12h	4.67	
08/06/2016	13h	4.6	
08/06/2016	14h	4.55	
08/06/2016	15h	4.68	ON
08/06/2016	16h	5.16	ON
08/06/2016	17h	5.8	ON
08/06/2016	18h	6.37	ON
08/06/2016	19h	6.79	ON
08/06/2016	20h	6.92	ON
08/06/2016	21h	6.52	ON
08/06/2016	22h	5.87	ON
08/06/2016	23h	5.5	
09/06/2016	0h	5.3	
09/06/2016	1h	5.05	
09/06/2016	2h	4.89	
09/06/2016	3h	4.51	
09/06/2016	4h	4.09	
09/06/2016	5h	3.17	
09/06/2016	6h	2.56	
09/06/2016	7h	1.58	
09/06/2016	8h	0.71	
09/06/2016	9h	0.3	
09/06/2016	10h	-0.11	
09/06/2016	11h	0.33	
09/06/2016	12h	0.1	
09/06/2016	13h	-0.08	
09/06/2016	14h	-0.22	
09/06/2016	15h	0.17	ON
09/06/2016	16h	0.57	ON
09/06/2016	17h	2.77	ON
09/06/2016	18h	4.35	ON
09/06/2016	19h	4.77	ON
09/06/2016	20h	4.94	ON
09/06/2016	21h	4.63	ON
09/06/2016	22h	4.42	ON
09/06/2016	23h	4.17	
10/06/2016	0h	3.99	
10/06/2016	1h	3.92	
10/06/2016	2h	3.54	
10/06/2016	3h	3.11	

10/06/2016	4h	3.2	
10/06/2016	5h	3.48	
10/06/2016	6h	3.65	
10/06/2016	7h	3.79	
10/06/2016	8h	3.93	
10/06/2016	9h	4.02	
10/06/2016	10h	4.19	
10/06/2016	11h	4.27	
10/06/2016	12h	4.5	
10/06/2016	13h	4.59	
10/06/2016	14h	4.64	
10/06/2016	15h	4.89	ON
10/06/2016	16h	5.35	ON
10/06/2016	17h	5.98	ON
10/06/2016	18h	5.86	ON
10/06/2016	19h	6.25	ON
10/06/2016	20h	6.07	ON
10/06/2016	21h	5.8	ON
10/06/2016	22h	5.54	ON
10/06/2016	23h	5.46	
11/06/2016	0h	5.5	
11/06/2016	1h	5.45	
11/06/2016	2h	5.68	
11/06/2016	3h	5.8	
11/06/2016	4h	5.87	
11/06/2016	5h	5.96	
11/06/2016	6h	5.99	
11/06/2016	7h	6.07	
11/06/2016	8h	6.11	
11/06/2016	9h	5.94	
11/06/2016	10h	6.11	
11/06/2016	11h	6.04	
11/06/2016	12h	6.18	
11/06/2016	13h	6.21	
11/06/2016	14h	6.21	
11/06/2016	15h	6.31	ON
11/06/2016	16h	6.46	ON
11/06/2016	17h	6.58	ON
11/06/2016	18h	7.07	ON
11/06/2016	19h	7.19	ON
11/06/2016	20h	8.23	ON
11/06/2016	21h	6.81	ON
11/06/2016	22h	5.03	ON
11/06/2016	23h	4.07	

86

Supplemental Table S3. Oligos used in the study.

**Oligos for Reverse transcription and qPCR**			
Target	Primer name	Sequence 5'-3'	For RT
At5g25760 control	UBC_qPCR_F	CTGCGACTCAGGGAATCTTCTAA	
	UBC_qPCR_R	TTGTGCCATTGAATTGAACCC	Y
At1g13320 control	PP2A QPCR F2	ACTGCATCTAAAGACAGAGTTCC	
	PP2A QPCR R2	CCAAGCATGGCCGTATCATGT	Y
At5g10140	FLC_4265_F (spliced sense)	AGCCAAGAAGACCGAACTCA	
	FLC_5683_R (spliced sense)	TTTGTCCAGCAGGTGACATC	Y
At5g10140	FLC_3966_F (unspliced sense)	CGCAATTTTCATAGCCCTTG	
	FLC_4135_R (unspliced sense)	CTTTGTAATCAAAGGTGGAGAGC	
	FLC unspliced RT (4029)	TGACATTTGATCCCACAAGC	Y
Total *COOLAIR* for field	set6_new_JH_LP	TGCATCGAGATCTTGAGTGTATG T	Y
	set6_new_JH_RP	ACGTCCCTGTTGCAAAATAAGC	
Total *COOLAIR* for TEX	Total_COOLAIR_ RP	CGCGCAGAGAGAGAGAGAG	
	Total_COOLAIR_L P	AAAACTTGTGTTTGCTTCACAG	Y
Class I *COOLAIR*	set2_new_2_LP	CCTGCTGGACAAATCTCCGA	Y
	set2_new_2_RP	TCACACGAATAAGGTGGCTAATT AAG	
Class II.i *COOLAIR*	Class II.i-F	CCTTTTCCTTACCTGGGTTTTCAT TTG	
	Class II.i-R	CTCACACGAATAAGAAAAGTAAA AGAGC	
ClassII/Dista l *COOLAIR*	Set4-new-F-195	GTATCTCCGGCGACTTGAAC	
	Set4-new-R-195	CGGATGCGTCACAGAGAACAG	
	FLC-158-F (RT primer for distal *COOLAIR*)	GCCCGACGAAGAAAAAGTAG	Y
**Oligos for ChIP at *COOLAIR* promoter region**			
ChIP-P1	ChIP-P1_LP	CCGGTTGTTGGACATAACTAGG	
	ChIP-P1_RP	CCAAACCCAGACTTAACCAGAC	
ChIP-P1	ChIP-P2_R	CGTGTGAGAATTGCATCGAG	
	ChIP-P2_F	AAAAACGCGCAGAGAGAGAG	

Supplemental Table S4. Oligos used for the 5’RACE assay.

**Target**	**Primer name**	**Sequence 5’-3’**	**Note**
COOLAIR	RT-R	GCCCGACGAAGAAAAAGTAG	For RT reaction
	R1	GTATCTCCGGCGACTTGAAC	Paired with ADP-F1, for first round PCR
	R2	AGTCACCTTCTCCAAACGTCG	Paired with ADP-F2, for second round PCR
	R3	GCAAGCTCTACAGCTTCTCCTC	Paired with ADP-F2, for second round PCR
TUB6	RT-R	ACACACCTTGAAGACAGTCGCA	For RT reaction
	R1	CAGCATCAATAAGCTCAGCTCCT	Paired with ADP-F1, for first round PCR
	R2	ACCAGGCTCAAGATCCATGAGA	Paired with ADP-F2, for second round PCR
5’ RACE adapter	ADP-F1	GCTGATGGCGATGAATGAACACTG	Paired with R1, for first round PCR
	ADP-F2	CGCGGATCCGAACACTGCGTTTGCTGGCTTTGATG	Paired with R2, for first round PCR
